# Glycosylation of β1 subunit plays a pivotal role in the toxin sensitivity and activation of BK channels

**DOI:** 10.1590/1678-9199-JVATITD-2020-0182

**Published:** 2021-06-02

**Authors:** Xiaoli Wang, Qian Xiao, Yudan Zhu, Hong Qi, Dongxiao Qu, Yu Yao, Yuxiang Jia, Jingkan Guo, Jiwei Cheng, Yonghua Ji, Guoyi Li, Jie Tao

**Affiliations:** 1 Institute of Biomembrane and Biopharmaceutics, Shanghai University, Shanghai, China.; 2 Department of Neurology and Central Laboratory, Putuo Hospital, Shanghai University of Traditional Chinese Medicine, Shanghai, China.; 3 Xinhua Translational Institute for Cancer Pain, Shanghai, China.; 4 Putuo Clinical Medical School, Anhui Medical University, Shanghai, China.

**Keywords:** BK channels, Glycosylation, β1-subunit, Toxin sensitivity, Kinetic property

## Abstract

**Background:**

The accessory β1 subunits, regulating the pharmacological and biophysical properties of BK channels, always undergo post-translational modifications, especially glycosylation. To date, it remains elusive whether the glycosylation contributes to the regulation of BK channels by β1 subunits.

**Methods:**

Herein, we combined the electrophysiological approach with molecular mutations and biochemical manipulation to investigate the function roles of N-glycosylation in β1 subunits.

**Results:**

The results show that deglycosylation of β1 subunits through double-site mutations (β1 N80A/N142A or β1 N80Q/N142Q) could significantly increase the inhibitory potency of iberiotoxin, a specific BK channel blocker. The deglycosylated channels also have a different sensitivity to martentoxin, another BK channel modulator with some remarkable effects as reported before. On the contrary to enhancing effects of martentoxin on glycosylated BK channels under the presence of cytoplasmic Ca^2+^, deglycosylated channels were not affected by the toxin. However, the deglycosylated channels were surprisingly inhibited by martentoxin under the absence of cytoplasmic Ca^2+^, while the glycosylated channels were not inhibited under this same condition. In addition, wild type BK (α+β1) channels treated with PNGase F also showed the same trend of pharmacological results to the mutants. Similar to this modulation of glycosylation on BK channel pharmacology, the deglycosylated forms of the channels were activated at a faster speed than the glycosylated ones. However, the V_1/2_ and slope were not changed by the glycosylation.

**Conclusion:**

The present study reveals that glycosylation is an indispensable determinant of the modulation of β1-subunit on BK channel pharmacology and its activation. The loss of glycosylation of β1 subunits could lead to the dysfunction of BK channel, resulting in a pathological state.

## Background

BK channels (voltage-dependent large-conductance Ca^2+^-activated K^+^ channels)，also known as Maxi-K channels, distributed in both excitable and non-excitable cells and are considered as key participants in a variety of physiological functions, including regulating smooth muscle tone[1-3], neuronal firing [4, 5], endocrine cell secretion [6, 7], cell proliferation [8, 9] and migration [10, 11]. Functional BK channels are a tetramer of four pore-forming α subunits encoded by a single gene Slowpoke (*Slo*) [12]. Different from the close homology of voltage-gated K^+^ (Kv) channel, the α subunit of BK channels possesses additional hydrophobic segments including a transmembrane helix (S0) which places the N terminus on the extracellular side of the plasma membrane [13, 14] and a long cytosolic C-terminal (S7-S10) where putative Ca^2+^-binding sites reside [14, 15]. Owing to the various tissue-specific β subunits, BK channels possess a rather complex diversity of subtype family. Four β subunit types have been characterized so far, β1 from smooth muscle[16], β2 from rat chromaffin cells[17, 18], β3 from testis [19, 20], and β4 from brain [21]. The regulatory β subunits are mainly responsible for modulating the BK channel kinetic behavior, Ca^2+^ sensitivity and pharmacological responses to their specific modulators, such as scorpion toxins [22-25].

Generally, extracellular loops of most membrane proteins, including ion channels, are N-glycosylated. Oligosaccharides attached to the nascent polypeptide by recognizing an iconic amino acid sequon (Asn-X-Ser/Thr). Glycosylation has been demonstrated to be crucial to the activity, intracellular trafficking and targeting, and cell surface expression of ion channels [26-28]. Inhibition of N-linked glycosylation altered the gating properties of Kv1.1[29], Kv1.2 [30, 31] and KvLQT/minK (I_sK_) [32]. N-linked glycosylation also increases the expression of Shaker potassium channels [33] and HERG channels on the cell surface [34]. Most significantly, some genetic disorders, represented by long QT (LQT) syndrome was found to be induced by the mutations of glycosylation sites in HERG channels [35]. A point mutation of glycosylation site (N604T) changed the inactivation and ion selectivity rather than plasma membrane expression of TRPV1 [36]. In addition, glycosylation could alter the conductance properties of sodium channels [37], the gating properties of rNav1.3 [38] and steady-state inactivation of Nav1.9 endogenously expressed in dorsal root ganglion neurons [39]. Such phenomenon also presents in auxiliary subunits of ion channels. Glycosylation of the sodium channel β4 subunit is developmentally regulated and participates in neuritic degeneration [40]. The inhibitory effect of iberiotoxin on BK channels was enhanced by deglycosylation on β4 subunits [41]. Deglycosylation of the β1-subunit changes the open probability (Po) of BK channels in murine colonic smooth muscle cells [42]. It was also reported that there are 3 N-glycosylated sites (N-X-T/S at N88, N96 and N119) located at the β2 subunits of BK channels. Deglycosylation of these sites could regulate gating kinetics, outward rectification and toxin sensitivity of BK (α+β2) channels [43].

Given the clues mentioned above, glycosylation, either through α or β subunit, may probably participate in regulating the overall performances of channels in response to the dynamic and complicated changes in physiological conditions. Yet, the subtle but significant variations in channel conformation and extracellular environment may bring out uncertainties to elucidate the role of glycosylation in BK channel function. On the other hand, there remain questions on how specific ligands of BK channels, such as iberotoxin and martentoxin behave on deglycosylated BK channels, which needs to be considered in realizing the molecular interactions between toxins and channels. It also needs to be clarified how oligosaccharide chains on β1 influence the kinetic properties of BK channels. To these ends, this study will provide a possible explanation on these questions.

## Methods

### Construction of channel mutants

The plasmids containing hSloα (U23767) and β1 (KCNMB1; U25138) were gifts from N.W. Davies (University of Leicester) and J.D. Lippiat (Leeds university) [44]. We used the KOD mutagenesis kit (Toyobo, Japan) to make point mutations in the β1 subunit. In brief, PCR reactions were performed by using the wild-type β1 as the template and a pair of complementary mutagenesis primers. The primers were designed with Primer 5.0 (PremierBiosoft, USA). The PCR products were used to transform competent bacterial cells (DH5α, Tiangen, China) to amplify the mutant plasmid of hSloα+β1. All mutant constructs were verified by sequencing (Lifetechnologies, China).

### Cell culture and transfection

All experiments were performed on HEK 293T cell lines. HEK 293T cells were obtained from Shanghai cell bank of Chinese Academy of Science. The cells were both cultured in Dulbecco’s modified Eagle medium (DMEM; Life Technologies, Grand Island, NY) supplemented with 10% heat-inactivated fetal bovine serum (FBS; Gibco, Grand Island, NY). Culture dishes were incubated at 37 °C in a humidified atmosphere containing 5% CO_2_, and subcultured approximately every 2-3 days. One day before transfection, HEK 293T cells were transferred to 24 well plates. At 90% confluence, cells were transiently transfected using Lipofectamine-2000 (Invitrogen, USA) at a ratio of 2 µL reagent with 1 µg total plasmid per well. Electrophysiological experiments and western blot analysis were performed at 1-2 days after transfection.

### Western blot analysis

Cells were harvested 48 hours after transfection, washed twice with ice-cold phosphate-buffered saline (PBS) and lysed with cell lysis buffer on ice for 30 min. The cell lysates were centrifuged for 15 min at 14,000×g at 4 °C and the protein concentration of the supernatant was determined. Equivalent amounts of total protein (40 µg/lane) were separated by 12% sodium dodecyl sulfate-polyacrylamide gel electrophoresis (SDS-PAGE), and then transferred to poly (vinylidene fluoride) membranes (milipore，USA) using the submerged method. The membranes were incubated with rabbit anti-human IgG at room temperature for 2 h following the incubation of the Maxi-K beta antibody (diluted 1:200, Alomone Labs Ltd., Israel) overnight then washed with phosphate buffered saline plus tween-20 (PBST, tween-20 0.05%), and incubated for 1 h at room temperature with a horseradish peroxidase-conjugated secondary antibody (goat anti-rabbit IgG, 1:10000, Abcam, UK). Blots were visualized with Chemi Doc^TM^ XRS+ Imaging System (Bio-RAD, U.S.A).

### Electrophysiological recordings

Whole-cell voltage-clamp experiments were performed following the procedures described previously [45], using an EPC-9 amplifier (HEKA Eletronik, Germany) at room temperature (21-25 °C). Patch pipettes were fabricated from glass capillary tubes by PC-10 Puller (Narishige, Japan) with the resistance of 2-3 MΩ. Data acquisition and stimulation protocols were controlled by a Pentium III computer (Legend, Beijing, China) equipped with Pulse/PulseFit 8.3 software (HEKA Eletronik, Germany). Capacitance transients were cancelled. Cells with a seal resistance (Rseal) below 1 GΩ were omitted. Series resistance (Rs) was compensated (80-85%) to minimize voltage errors, and cells with an uncompensated series resistance (Rs) above 10 MΩ were omitted. Leak subtraction was performed using P/6 protocol. Data were low-passed at 10 kHz. The rate of solution exchange was studied using solutions with different KCl concentrations and found to be about 95% complete within 20 s. Unless stated specially, For HEK 293T cells, the holding potential was -80 mV. All the recordings were performed with the pulse of +100 mV.

### Solutions and drugs

In the patch-clamp recordings, the standard bath solution for HEK 293T cells was consisted of the following components (in mM): NaCl 135, KCl 5, MgCl_2_ 1.2, CdCl_2_ 2.5, HEPES 5, glucose 10 (pH 7.4 with NaOH). Pipette solutions for HEK 293T cells were composed of the following components (in mM): NaCl 10, KCl 117, MgSO_4_ 2, HEPES 10, MgATP 2, EGTA 1 (pH 7.2 with KOH). The total Ca^2+^ to be added to give the desired free concentration was calculated using the program Maxchelator (http://www.stanford.edu/%7Ecpatton/maxc.html).

In the western blot analysis, the phosphate-buffered saline (PBS) was consisted of the following components (in mM): NaCl 135, KCl 4.7, Na_2_HPO_4_ 10, NaH_2_PO_4_ 2 (pH = 7.4). 

BmK venom collected by electric stimulation was purchased from a scorpion farm in Zhengzhou, Henan province, China. Martentoxin was purified according to the method described by Ji [46].

The toxins were dissolved in the bath solution, supplemented with 1 mg/ml bovine serum albumin (BSA) in order to prevent adherence of the toxin to the vials and the perfusion apparatus. Application of 1 mg/ml BSA alone did not alter BK channel function. Unless otherwise stated, all reagents were purchased from Sigma.

### Data analysis

Data were analyzed by PulseFit 8.5 (HEKA Eletronik, Germany) and Origin 7.5 (Northampton, Massachusetts, USA). Results of data analysis were expressed as mean ± S.E.M. and n represents the number of the cells examined. 

The Statistical significance was determined using the unpaired Student’s t-Test or one-way ANOVA, and an asterisk denotes P<0.05 unless otherwise stated. The degree of toxin effect was calculated by expressing the remaining current after each drug exposure as a fraction of the current magnitude of the patch prior to the first drug exposure (i.e., fractional current remaining, I_f_). 

BK channel (α+β1) currents were elicited by the step pulses ranging from -50 to +120 mV for 200 ms with the increments of 10 mV. The holding potentials were held at -80 mV for BK channel (α+β1). Current density calculation formula (pA/pF), where pA represents the current of BK channel (α+β1) and pF represents the membrane area of measured cell. For determining the voltage dependence of activation, the conductance was calculated using the formula: G(V)= I(V)/(V-ErK), where I(V) is the current of BK channel (α+β1) at the command voltage V, and ErK is the reversal potential. The conductance was normalized to the maximal value and the voltage dependence for activation of BK channel (α+β1) fitted to a Boltzmann equation: f(x) = -1/(1+exp((x-V_1/2_)/k))+1, where V_1/2_ is the voltage at which half-maximal activation occurs, and k describes the slope of the fit.

The activation kinetics was analyzed by fitting the decay course of the BK channel currents to a single exponential function: f(t)= C+A×exp[-(t-t_0_)/τ], where t is time, t_0_ is the time when the currents were just starting to exponentially increase, A represents the amplitudes of the channels activating with the time constant τ and C is the steady-state asymptote approximating to the maximum value of the current amplitude (I_max_).

## Results

### Glycosylation of β1 co-expressed with hSloα subunit in HEK293T cells

**Figure 1.**
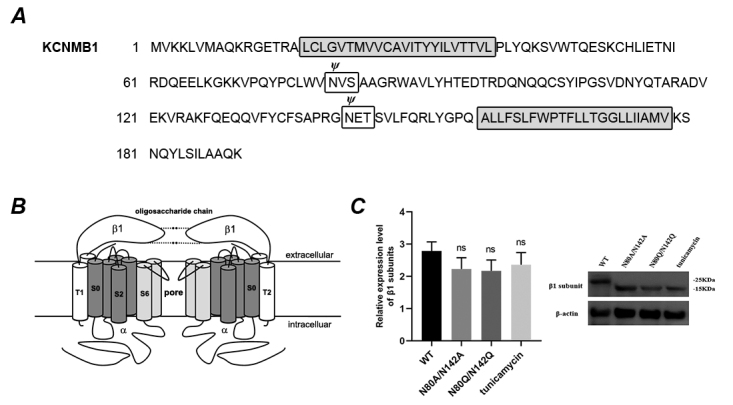
Molecular properties of BK channel β1-subunit. **(A)** The primary structure of a subunit protein, including N-glycosylation site (white boxes) and transmembrane segment (gray boxes). **(B)** Model of BK (α+β1) channels (two of the four subunits are shown), including segments of α subunits, extracellular loop of β1 and oligosaccharide chains on β1 (dotted line). **(C)** Western blot showing β1-subunit. Molecular mass of the ~25 kDa immunoreactive band was wild-type β1-subunit (lane 1). Immunoreactive protein, with a molecular mass of ~20 kDa was double glycosylation site mutant β1 N80A/N142A (lane 2) and β1 N80Q/N142Q (lane 3). The immunoreactive band with a molecular mass of ~20 kDa on lane 4 was the β1-subunit by treatment with tunicamycin. There was no significant difference among the wild-type β1 subunit, N80A/N142A mutant, N80Q/N142Q mutant and the tunicamycin treated group in protein relative expression level (p ＞ 0.05).

We designed 4 pairs of complementary mutagenesis primers to construct the N80A/N142A mutant and N80Q/N142Q mutant of the β1 subunit of the BK channel, which were identified by sequencing (Additional file 1, 1A-1C). To confirm whether β1-subunit expressed in the HEK 293T cells was glycosylated, the western blotting was used to detect the molecular weight of β1 mutations (N80A/N142A and N80Q/N142Q, Figure 1C) before the whole experiment. Both lysates from wild type (β1)-expressed cells and double mutations (β1 N80A/N142A and β1 N80Q/N142Q)-expressed cells were assayed with antibody specific for BK β1-subunit. A ~25 kDa band observed here represents the wild-type β1-subunit (Figure 1C, first lane), which is consistent with the previous report [42]. The double mutants of glycosylation sites (N80A/N142A) were detected with a smaller molecular mass of ~20 kDa (Figure 1C, second lane). Similar result was also observed from the analysis for another double mutant (N80Q/N142Q) (Figure 1C, third lane). Different mutations (N80A/N142A and N80Q/N142Q) were constructed to rule out the significant alteration of β1 molecular weight and confirm the removal of oligosaccharide from the β1 glycosylation sites. Furthermore, tunicamycin, a potent inhibitor of N-linked glycosylation, was employed [38, 41] to make sure that deglycosylation on β1-subunit was achieved successfully by site-directed mutation. 30 min after HEK293T cells were transfected with the hSloα+β1 (wild-type), tunicamycin at the final concentration of 10 μM was added and the cells were grown under the presence of tunicamycin for 15 h. Cell lysates were assayed by western blot. Wild-type β1-subunit from tunicamycin-treated cells also has a same molecular weight of ~20 kDa (Figure 1C, forth lane) as the double mutant (β1 N80A/N142A or β1 N80Q/N142Q). This indicated that β1 is glycosylated in HEK 293T cells. N80 and N142 were the correct N-linked glycosylation sites in β1-subunit. Western blotting and electrophysiological assay showed that there were no significant difference in protein relative expression level and current density between the wild-type β1 subunit, the two double mutants and the tunicamycin treated group (Figure 1D and Additional file 1D). In other words, β1 subunit mutants or the use of glycation inhibitors did not lead to changes in expression levels.

### Deglycosylation of β1 changing the toxin sensitivity of BK channel (α+β1)

**Figure 2.**
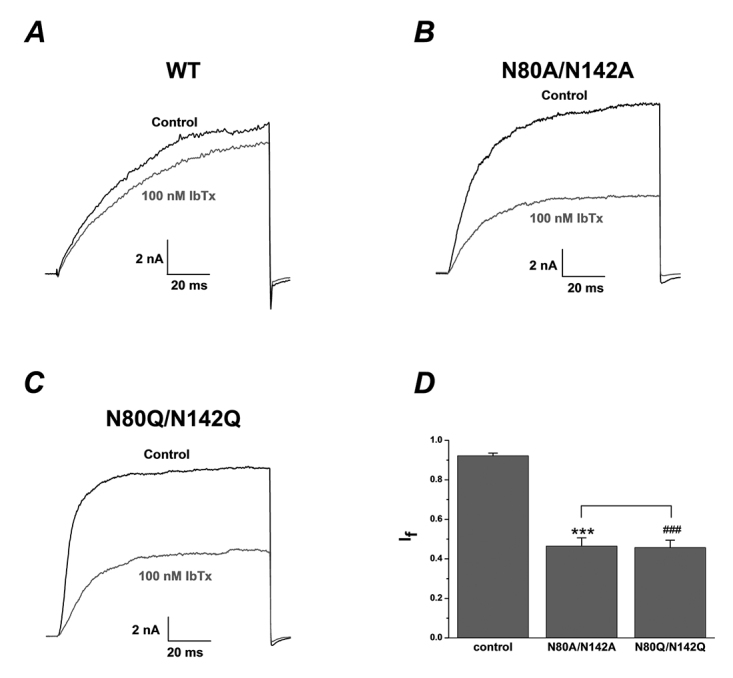
The inhibition effect of iberiotoxin on BK channels (a+β1) could be enhanced after deglycosylation of β1 subunit. **(A)** Representative whole cell current traces from HEK 293T cells expressing wild-type BK channels (a+β1) before and after the application of IbTx 100 nM. The holding voltage was -80 mV and the currents were elicited by a pulse of +100 mV in the presence of 300 nM free Ca^2+^ in the pipette solution. **(B)** Representative whole cell current traces from HEK 293T cells expressing mutant BK channels N80A/N142A before and after the application of IbTx 100 nM. **(C)** Representative whole cell current traces from HEK 293T cells expressing mutant BK channels N80Q/N142Q before and after the application of IbTx 100 nM. **(D)** Statistical analysis of pharmacological modulation of wild-type BK channels (a+β1) (n = 7) and N80A/N142A (n = 7, ***p ＜ 0.001) or wild-type BK channels (a+β1) (n = 7) and N80Q/N142Q (n = 6, ^###^p ＜ 0.001) by IbTx 100 nM. The inhibition ratios of IbTx on the currents of N80A/N142A and N80Q/N142Q were not significantly different (p ＞ 0.05).

To assess the pharmacological sensitivity of deglycosylated BK channels, wild type and mutant BK (α+β1) channels were expressed in HEK293T cells. The cells transfected with wild type and mutant BK (α+β1) channels were pre-treated with 200 nM thapsigargin, an irreversible inhibitor of the sarcoplasmic reticulum Ca^2+^-ATPase pump for 30 min at 37 °C in order to deplete intracellular Ca^2+^ stores [47] before performing whole cell patch recordings. When the free Ca^2+^ concentration in the pipette solution was buffered to 300 nM, the currents of BK (α+β1) channel evoked by +100 mV pulse could not be effectively blocked by iberiotoxin, a specific and potent blocker of BK channels to hSloα [21], even at the concentration of 100 nM (I_f_: 0.92 ± 0.01, n = 7, Figure 2A). However, the double mutant (β1 N80A/N142A) does not protect the channel against iberiotoxin at this concentration (I_f_: 0.46 ± 0.04, n = 7, Figure 2B, p ＜ 0.001). In addition, another double glycosylation site mutant β1 (β1 N80Q/N142Q) also showed high sensitivity to iberiotoxin (I_f_: 0.46 ± 0.04, n = 6, Figure 2C, p ＜ 0.001). As summarized in Figure 2D, both double mutants N80A/N142A and N80Q/N142Q had significant difference of the sensitivity to the inhibition of 100 nM iberiotoxin from wild-type BK (α+β1) channels. It should be noted here that two double mutants don’t have any obvious difference of the sensitivity to iberiotoxin. 

**Figure 3.**
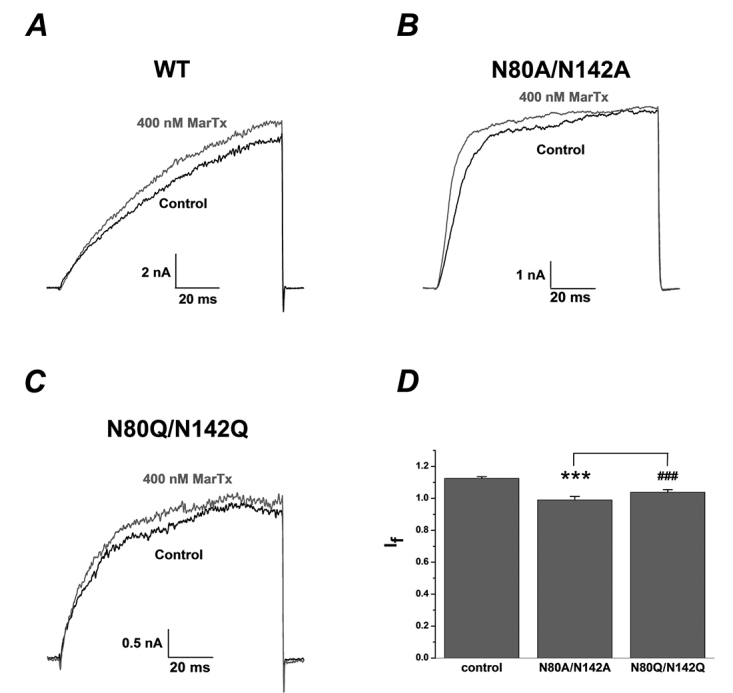
Deglycosylation of β1 subunit removed the enhancement effect of martentoxin on BK channels (α+β1). **(A)** Representative whole cell current traces from HEK 293T cells expressing wild-type BK channels (α+β1) before and after the application of martentoxin 400 nM. The holding voltage was -80 mV and the currents were elicited by a pulse of +100 mV in the presence of 300 nM free Ca2^+^ in the pipette solution. **(B)** Representative whole cell current traces from HEK 293T cells expressing mutant BK channels N80A/N142A before and after the application of martentoxin 400 nM. **(C)** Representative whole cell current traces from HEK 293T cells expressing mutant BK channels N80Q/N142Q before and after the application of martentoxin 400 nM. **(D)** Statistical analysis of pharmacological modulation of wild-type BK channels (α+β1) (n = 6) and N80A/N142A (n = 5, ***p ＜ 0.001) or wild-type BK channels (α+β1) (n = 6) and N80Q/N142Q (n = 6, ###p ＜ 0.001) by martentoxin 400 nM. The effect ratios of martentoxin on the currents of N80A/N142A and N80Q/N142Q were not significantly different (p ＞ 0.05).

To investigate this pharmacological modulation of BK channels by deglycosylation further, martentoxin, a specific modulator of BK channel subtypes was also used here. Martentoxin can significantly increase the activity of the BK channel (α+β1) subtype with an EC_50_ of 495 nM [48]. Under the same condition as above, the evoked currents of BK (α+β1) channels were increased by martentoxin at the concentration of 400 nM (I_f_: 1.13 ± 0.01, n = 6, Figure 3A). In contrast to this enhancement of glycosylated channel activity by martentoxin, both the double mutants N80A/N142A and N80Q/N142Q were insensitive to 400 nM martentoxin (I_f_: 0.99 ± 0.02, n = 5, p ＜ 0.001, Figure 3B; I_f_: 1.04 ± 0.02,n = 6, p ＜ 0.001, Figure 3C), And there were no significant differences between N80A/N142A and N80Q/N142Q of their sensitivity to martentoxin (Figure 3D).

**Figure 4.**
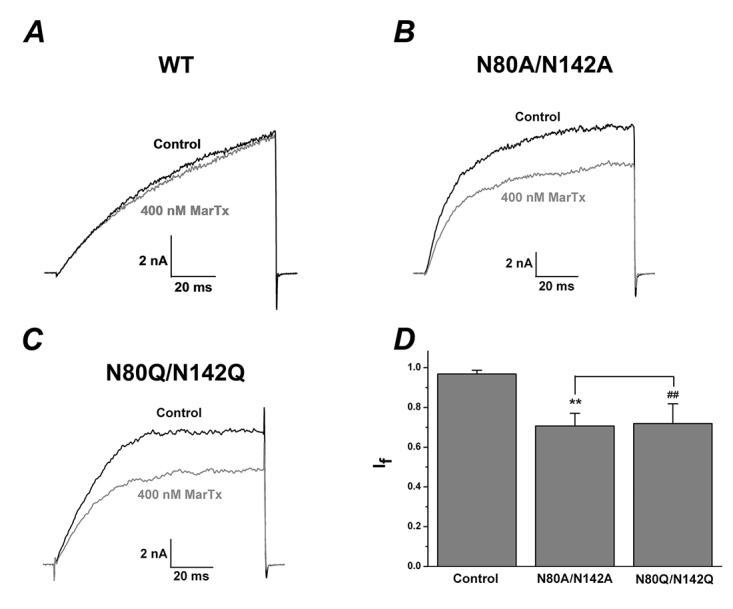
Deglycosylation of β1 subunit altered the pharmacological effect of martentoxin on BK channels (α+β1) in the absence of cytoplasmic Ca^2+^. **(A)** Representative whole cell current traces from HEK 293T cells expressing wild-type BK channels (α+β1) before and after the application of martentoxin 400 nM. The holding voltage was -80 mV and the currents were elicited by a pulse of +100 mV. **(B)** Representative whole cell current traces from HEK 293T cells expressing mutant BK channels N80A/N142A before and after the application of martentoxin 400 nM. **(C)** Representative whole cell current traces from HEK 293T cells expressing mutant BK channels N80Q/N142Q before and after the application of martentoxin 400 nM. **(D)** Statistical analysis of pharmacological modulation of wild-type BK channels (α+β1) (n = 3) and N80A/N142A (n = 3, **p ＜ 0.01) or wild-type BK channels (α+β1) (n = 3) and N80Q/N142Q (n = 4, ##p ＜ 0.01) by martentoxin 400 nM. The inhibition ratios of martentoxin on the currents of N80A/N142A and N80Q/N142Q were not significantly different (p ＞ 0.05).

When the pipette solution contained 10 mM EGTA without any Ca^2+^, the channels could only be activated by voltage. The currents of glycosylated BK (α+β1) channels could not be modulated by martentoxin at 400 nM (I_f_: 0.97 ± 0.018, n = 3, Figure 4A). In contrast, the currents of double mutants N80A/N142A and N80Q/N142Q could be potently inhibited by 400 nM martentoxin (N80A/N142A I_f_: 0.71 ± 0.064, n = 3, p ＜ 0.001; N80Q/N142Q I_f_: 0.72 ± 0.099, n = 4, p ＜ 0.001, Figures 4B and 4C). The inhibition ratios of martentoxin on the currents of N80A/N142A and N80Q/N142Q were not significantly different (Figure 4D). 

### Deglycosylation of β1 modifying the activation kinetics of BK channel (α+β1) in HEK293T cells

**Figure 5.**
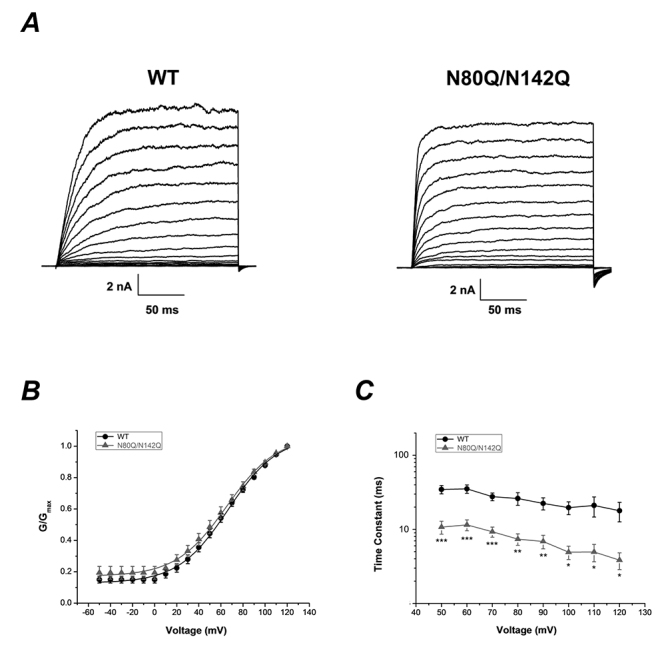
Deglycosylation of β1 subunit did not shift steady-state activation of BK channels (α+β1). **(A)** Representative whole cell current traces from HEK 293T cells expressing wild type BK channels (α+β1) (left), N80Q/N142Q (right). The channels were stepped from -50 mV to +120 mV in 10 mV steps with the holding potential at -80 mV. **(B)** The plots of the normalized conductance fit well with Boltzmann function. The voltage-dependent activation curves were insignificantly shifted before and after the deglycosylation. **(C)** Time constants are shown for wild-type BK channels (α+β1) (n = 5, black circle), N80Q/N142Q (n = 7, gray circle).

It has been well-established that the gating properties of BK channel depend largely on the interactions between α and β subunits [19, 21, 49-51]. Therefore, the subtle alterations in structure caused by glycosylation would have potential impact on the gating kinetics of BK channel (α+β1). To test this hypothesis, the voltage-dependent activation of the deglycosylated β1 mutants (β1 N80A/N142A and N80Q/N142Q) was examined. HEK293T cells transfected with wild-type or mutant BK (α+β1) channels were also pre-treated with 200 nM thapsigargin for 30 min at 37 °C. The free Ca^2+^ concentration in the pipette solution was also controlled to 300 nM in the patch clamp recording. The BK (α+β1) currents were elicited by the step pulses ranging from -50 to +120 mV for 200 ms with the increments of 10 mV. The activation curve of the double glycosylation site β1 mutant (β1 N80Q/N142Q) as well as the half-maximal voltage (V_1/2_) of activation was not significantly shifted compared to the wild-type BK (α+β1) channels (WT: V_1/2_ = 66.52 ± 1.47,n = 9, p ＞ 0.05; β1 N80Q/N142Q: V_1/2_ = 65.35 ± 1.48, n = 10, p ＞ 0.05, Figure 5B and Table 1). 

**Table 1.** Effect of deglycosylation on V_1/2_ and slope of conductance vs. voltage curves.

Wild-type and mutants	V_1/2_ , mV	Slope , mV	n
Wild type	66.52 ± 1.47	22.55 ± 1.21	9
N80Q/N142Q	65.35 ± 1.48	22.89 ± 1.24	10

The groups of β1 wild type and mutants have no significant difference, at the level of 0.05, tested by one-way ANOVA.

To evaluate the influence of deglycosylation on activation rate, we compared the activation time constants between wild-type and mutant channels. Time constants of activation at each potential were determined by fitting each current with a single exponential equation (in Materials and Methods, Data analysis). When the whole cell current tracings were evoked at each potential from +50 mV to +120 mV for wild-type BK (α+β1) channels and mutant channels, it was observed that the activation time constants was remarkably decreased in β1 N80Q/N142Q mutants compared to that of wild-type channels (n = 7, p<0.001 from +50 to +70 mV, p<0.01 from +80 mV to +90 mV, p<0.05 from +100 mV to +120 mV, Figure 5C).

### The inhibitory effects of IbTX and MarTX on BK channels (α+β1) were detected by exerting glycosidase

**Figure 6.**
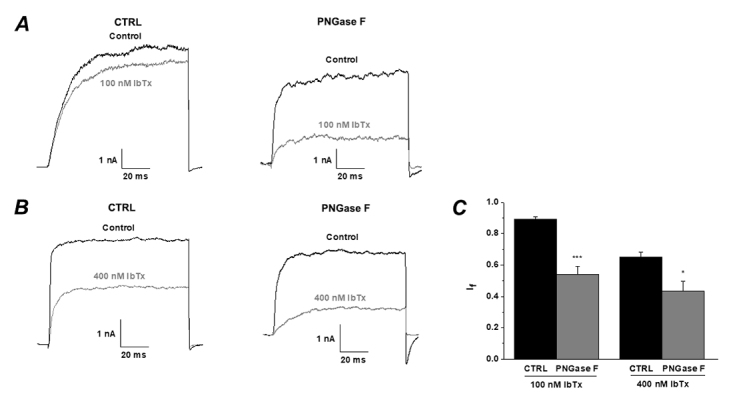
The application of PNGase F enhances the inhibitory effects of IbTX on BK (α+β1) channel. **(A-B)** Whole-cell current traces from deglycosylation BK channels before and after the application of IbTx **(A)** 100 nM and **(B)** 400 nM without treatment (left, CTRL) or pretreatment with PNGase F (right, PNGase F). The holding voltage was -80 mV and the currents were elicited by a pulse of +100 mV. **(C)** Statistical analysis of the inhibitory ratios of IbTX on BK (α+β1) channel without treatment (left, CTRL) or pretreatment with PNGase F.

**Figure 7.**
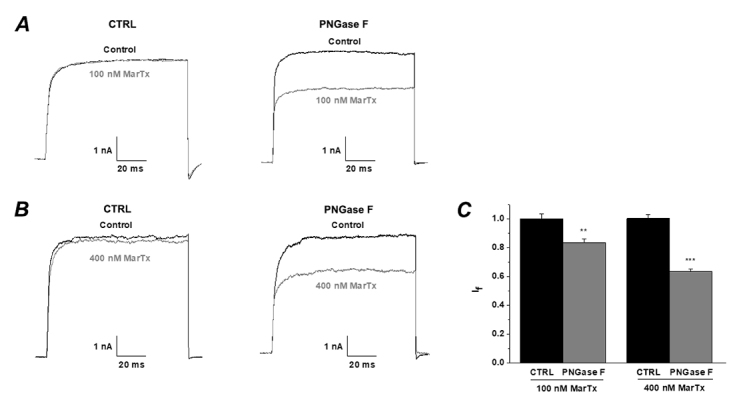
The application of PNGase F enhances the inhibitory effects of MarTX on BK (α+β1) channel. **(A-B)** Whole-cell current traces from deglycosylation BK channels before and after the application of MarTX **(A)** 100 nM and **(B)** 400 nM without treatment (left, CTRL) or pretreatment with PNGase F (right, PNGase F). The holding voltage was -80 mV and the currents were elicited by a pulse of +100 mV. **(C)** Statistical analysis of the inhibitory ratios of MarTX on BK (α+β1) channels without treatment (left, CTRL) or pretreatment with PNGase F.

In order to exclude the structural changes induced by a single or double amino acid mutation in β1 subunits, which affect the activity of channels, wild type of BK (α+β1) channels were expressed in HEK293T cell. The cells transfected with wild type BK (α+β1) channels were treated with PNGase F (1000 units) for 1 hour at 37 °C so that PNGase F could remove the N-glycosylation of β1 subunits before performing whole cell patch recordings. When the free Ca^2+^ concentration in the pipette solution was buffered to 300 nM, the currents of BK (α+β1) channel, pre-treated with PNGase F, evoked by +100 mV pulse could be effectively blocked by iberiotoxin at the concentration of 100 nM and 400 nM (Figure 6A and 6B). Statistical analysis showed that pre-treated with PNGase F, the inhibition of both concentrations of IbTX on BK (α+β1) channel had been significantly enhanced (100 nM IbTX CTRL group: I_f_ = 0.89 ± 0.01vs. 100 nM IbTX PNGase F group: I_f_ = 0.54 ± 0.05; 400 nM IbTX CTRL group: I_f_ = 0.65 ± 0.03 vs. 400 nM IbTX PNGase F group: I_f_ = 0.43 ± 0.06, Figure 6C). When the pipette solution without any Ca^2+^, the BK channel (α+β1) is activated only by voltage. The currents of BK (α+β1) channels could be obviously inhibited by MarTX (Figure 7). Statistical analysis showed that inhibitory ratio of the PNGase F group was more pronounced than that of control group (100 nM MarTX CTRL group: I_f_ = 1.00 ± 0.03vs. 100 nM MarTX PNGase F group: I_f_ = 0.83 ± 0.03; 400 nM MarTX CTRL group: I_f_ = 1.00 ± 0.03 vs. 400 nM MarTX PNGase F group: I_f_ = 0.63 ± 0.02, Figure 7C).

## Discussion

BK channels (α+β1) are mainly distributed in smooth muscle, hair cells and some of the neurons, which are often considered as the direct target of many natural products. Researches on the role of N-linked glycosylation of β subunit may contribute to the understanding of the molecular mechanisms underlying the interaction of BK channels with specific ligands or auxiliary subunits as well as further reveal physiological functions of BK channels in cells and organs.

### Regulation on the pharmacological characterization of BK channel subtypes by deglycosylation of β-subunit

β-subunit usually changes the pharmacological property of BK channels, which is different from that of other potassium channels. β1-subunit co-expressed with the BK channel could increase the sensitivity to charybdotoxin, one of blockers for BK channel subtypes, whereas the BK channels formed by α and β4 subunits (BK (α+β4) channels) become insensitive to charybdotoxin [21, 23]. Another BK channel blocker, iberiotoxin, could potently inhibit the currents of BK channels which consist of α subunit alone. Co-expressed with either β1 or β4 subunits, BK channel subtypes become insensitive to iberiotoxin, respectively [21]. Similar phenomenon could also be seen for the BK channels modulated by slotoxin, a polypeptide purified from the venom of *Centruroides noxius* [52]. 

As an extracellular part of β subunits, it is worthy of our consideration whether glycosylation is important to adjust the toxin sensitivity of BK channel subtypes, especially participating in influencing the interaction between channels and toxins. In the present study, BK channels co-expressed with deglycosylated β1 mutants (N80Q/N142Q) were found to be more sensitive to iberiotoxin than that of wild type β1. Actually, such phenomenon is also found in deglycosylated β4 subunit. Deglycosylated β4 becomes less effective than wild type in protecting the channel against the block by iberiotoxin [41]. The results illuminated that oligosaccharide chains on the extracellular loop of β subunit might act as a shield to prevent toxins binding to the BK channel pore. However, some evidences supported another possibility that the removal of the oligosaccharides caused a conformational change in the interaction between α and β subunit. When BK (α+β4) channels were pretreated with martentoxin, the subsequent iberiotoxin acted more potently to inhibit the BK currents than applied it alone [53]. When three basic residues (Lys-120, Arg-121, and Lys-125) of β4 subunit were mutated, the affinity of charybdotoxin on BK channels could be significantly enhanced [54]. Oligosaccharides were not removed from BK channels in the reports above. It may allow us to infer that the deglycosylation on β subunits could alter the conformation of BK channels, the same effects as the pretreated toxin or site mutation. The conformational change increased binding affinities between BK channels and toxins.

As an effective tool to discriminate different BK channel subtypes, martentoxin was tailored to study the regulatory effects of glycosylation on BK channels. The current amplitude of wild-type BK (α+β1) channels could be increased by martentoxin. Interestingly, when co-expressed with non-glycosylated β1 (N80Q/N142Q or N80A/N412A), channels were found to be insensitive to martentoxin. In contrast, martentoxin has no significant effect on BK (α+β1) channels when intracellular free calcium was deprived. When two glycosylation sites (N80Q/N142Q or N80A/N412A) were mutated in β1 subunit, BK (α+β1) channels could be inhibited by martentoxin. The results suggest that the inhibitory sites of the BK (α+β1) channels for martentoxin were prevented by oligosaccharides.

### Modification on the kinetic properties of BK channels by deglycosylation of β1-subunit

β1 subunit increases the Ca^2+^ sensitivity of BK channels by altering the apparent affinity of the channels’ Ca^2+^ binding sites and slows down the activation kinetics of these channels. BK channels containing the α-subunits co-expressed with β1-subunit produce sustained currents that do not inactivate [49, 55].

Oligosaccharide chains, the ‘branched chain’ of β1-subunit, may regulate the kinetic properties of the BK channels. In the present study, the double glycosylation site mutants (β1 N80Q/N142Q) could remarkably accelerate the activation of BK channel compared to that of wild-type channels. In contrast, the activation time constants of single mutation for either β1 N80Q or N142Q mutant had no significant difference with wild-type channels. It could be infered that these two glycosylation sites on β1 subunit were synergically coupled in β1. The alteration of activation kinetics resulted from completely deglycosylation on β1 subunit. In another report, the open probability (P_o_) and mean open time of BK (α+β1) channels were both increased by deglycosylation of β1 [42]. The results demonstrated that deglycosylation on β1 could change the activation of BK channel, which might further influence the channel function directly. 

It is worth mentioning that the regulation of glycosylation on channel kinetics might be influenced by other environmental factors. In a previous report, deglycosylation of β1 could shift the voltage of steady-state activation toward more positive potentials, when Ca^2+^ concentration was controlled lower than 1 μM [42], which was different from our data (Figure 5B). In fact, the results are not contradictory possibly because of the following reasons: 1) Depend on calcium sensitivity of BK channels. The calcium sensitivity of BK channels formed by hsloα+β1 might be higher than the BK channels endogenously expressed in murine smooth muscle cells. The hsloα+β1 channels at low calcium concentration (300 nM) show the similar effects of murine BK channels at high calcium concentration (>1 μM) [42]. 2) Depend on membrane environment of BK channels. Unlike previous report, the current of BK (α+β1) channels was recorded by different patch clamping mode in present study. Consequently, the resulted midpoint voltage (V_1/2_) was somewhat different compared to the previous one, which could be explained by the subtle changes in mechanical state of the lipid membrane, which was closely related to gating properties [56]. 

### Physiological and pharmacological significance of glycosylation on β1 subunit of BK channels

BK (α+β1) channels activated by local Ca^2+^ release could regulate the membrane potential of arterial smooth muscle cells. Loss of the β1 subunit leads to hypertension, whereas a gain in β1 subunit function is associated with protection against hypertension in humans [57]. In present study, glycosylation on β1 subunits effectively protect BK channels against the regulation of modulators, which possibly implied an important role of glycosylation in maintaining blood pressure and the tone of vascular under physiological condition. On the other hand, deglycosylation on β1 subunits might have great significance in pathological conditions due to accelerating the activation as well as increasing open probability of BK channels, which makes the cell membrane tend to hyperpolarization and smooth muscles more relaxed. Deglycosylation will deserve to be considered as a target in the treatment of channelopathies and drug design. Fusion with glycosylase, specific peptides targeting BK channel might be selected as a therapeutic agent to mitigate the symptom of hypertension. Anyway, the realization of these ideas and resolution of mechanisms still need a large number of experiments in the future.

In summary, deglycosylation on β1 subunits increased the sensitivity to iberotoxin and martentoxin as well as accelerate the activation of BK (α+β1) channels. It would have important implications on the diversity of structure and function of BK channels. Especially at molecular level, in addition to key residues or important region on channel proteins, glycosylation become another indispensable factor for regulating the pharmacological properties of BK channel subtypes. 

## Conclusion

The present study investigated that deglycosylation of β1 subunits through double-site mutations (β1 N80A/N142A or β1 N80Q/N142Q) could significantly increase the inhibitory potency of iberiotoxin. Meanwhile, the deglycosylated channels also have a different sensitivity to martentoxin. On the contrary to the enhancing effects of martentoxin on glycosylated BK channels under the presence of cytoplasmic Ca^2+^, deglycosylated channels were not affected by the toxin. Surprisingly, the deglycosylated channels were inhibited by martentoxin under the absence of cytoplasmic Ca^2+^, while the glycosylated channels were not inhibited under this same condition. In addition, wild type BK (α+β1) channels were treated with PNGase F also showed the same trend of pharmacological results to the mutants. Similar to this modulation of glycosylation on BK channel pharmacology, the deglycosylated forms of the channels were activated at a faster speed than the glycosylated ones though the V_1/2_ and slope were not changed by the glycosylation. In a word, the present work reveals that glycosylation is an indispensable determinant of the modulation of β1-subunit on BK channel pharmacology and its activation. The loss of glycosylation of β1 subunits could lead to the dysfunction of BK channel, resulting in a pathological state.

### Abbreviations

BK channels: voltage-dependent large-conductance Ca2^+^-activated K^+^ channels; Kv channel: voltage-gated K^+^ channel; LQT: long QT syndrome; Rseal: seal resistance; Rs: series resistance; If: fractional current remaining; Imax: the maximum value of the current amplitude; V_1/2_: the half-maximal voltage; Po: the open probability.

## Supplementary material

The following online material is available for this article:

Additional file 1. The construction of N80A/N142A and N80Q/N142Q mutants of β1 subunit. **(A)** The complementary primer sequences were designed to construct N80A/N142A mutant and N80Q/N142Q mutant of β1 subunit. Primer 1 (S1, A1) and primer 2 (S2, A2) were used to construct N80A/N142A mutant, and primer 3 (S3, A3) and primer 4 (S4, A4) were used to construct N80Q/N142Q mutant. **(B)** Nucleic acid sequence alignment between wild-type, N80A/N142A and N80Q/N142Q mutants of β1 subunit. **(C)** Amino acid sequence alignment between wild-type, N80A/N142A and N80Q/N142Q mutants of β1 subunit. **(D)** Statistical analysis of current density of wild-type BK channels (α+β1) (n = 8) and N80A/N142A (n = 8, ns, p ＞ 0.05) or wild-type BK channels (α+β1) (n = 8) and N80Q/N142Q (n = 8, ns, p ＞ 0.05) or wild-type BK channels (α+β1) (n = 8) and BK (α+β1) channels pretreated with PNGase F (n = 8, ns, p ＞ 0.05).
