# Supplementary material for: Glycosylation of β1 subunit plays a pivotal role in the toxin sensitivity and activation of BK channels
Source: J Venom Anim Toxins Incl Trop Dis. 2021 Jun 2;27:e20200182. doi: 10.1590/1678-9199-JVATITD-2020-0182 (PMC8183112; doi:10.1590/1678-9199-JVATITD-2020-0182)
Supplement: Supplementary file 1 [file 1678-9199-jvatitd-27-e20200182-s1.pdf]

J Venom Anim Toxins incl Trop Dis
https://doi.org/10.1590/1678-9199-JVATITD-2020-0182

Supplementary Material to "Glycosylation of β1 subunit plays a pivotal role in the toxin sensitivity and activation of BK channels"

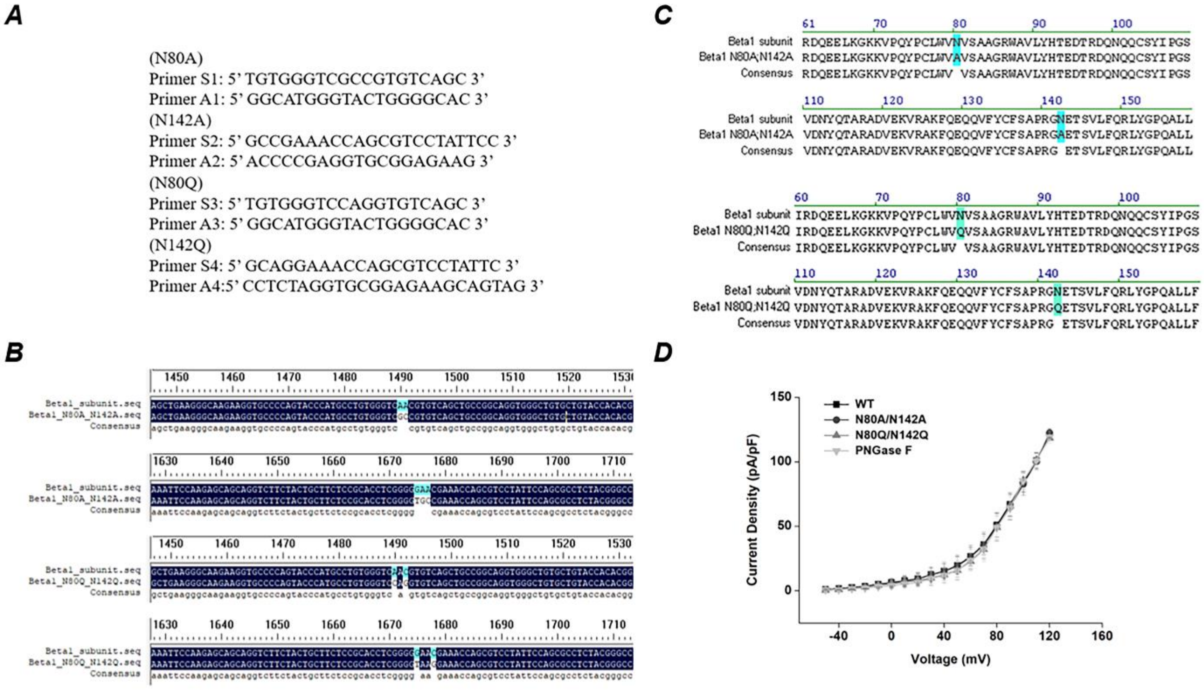

Additional file 1. The construction of N80A/N142A and N80Q/N142Q mutants of β1 subunit. **(A)** The complementary primer sequences were designed to construct N80A/N142A mutant and N80Q/N142Q mutant of β1 subunit. Primer 1 (S1, A1) and primer 2 (S2, A2) were used to construct N80A/N142A mutant, and primer 3 (S3, A3) and primer 4 (S4, A4) were used to construct N80Q/N142Q mutant. **(B)** Nucleic acid sequence alignment between wild-type, N80A/N142A and N80Q/N142Q mutants of β1 subunit. **(C)** Amino acid sequence alignment between wild-type, N80A/N142A and N80Q/N142Q mutants of β1 subunit. **(D)** Statistical analysis of current density of wild-type BK channels (α+β1) (n = 8) and N80A/N142A (n = 8, ns, $p > 0.05$) or wild-type BK channels (α+β1) (n = 8) and N80Q/N142Q (n = 8, ns, $p > 0.05$) or wild-type BK channels (α+β1) (n = 8) and BK (α+β1) channels pretreated with PNGase F (n = 8, ns, $p > 0.05$).
